# Supplementary material for: Mosaic anatomy in an early fossil squamate
Source: Nature. 2025 Oct 1;647(8090):673–9. doi: 10.1038/s41586-025-09566-y (PMC12629976; doi:10.1038/s41586-025-09566-y)
Supplement: Supplementary file 1 — Supplementary Discussion, containing: (A) Summary and taxonomic status of parviraptorid specimens (B) Summary description of Breugnathair elgolensis (NMS G.2023.7.1) and (C) Osteohistology. [file 41586_2025_9566_MOESM1_ESM.pdf]

nature portfolio

https://doi.org/10.1038/s41586-025-09566-y

Supplementary information

Mosaic anatomy in an early fossil squamate

In the format provided by the authors and unedited

Supplementary Discussion

Mosaic anatomy in an early fossil squamate

Roger B. J. Benson, Stig A. Walsh, Elizabeth F. Griffiths, Zoe Kulik, Jennifer Botha, Vincent Fernandez, Jason H. Head, Susan E. Evans

Table of Contents

Part A. Summary and taxonomic status of parviraptorid specimens
Part B. Summary description of *Breugnathair elgolensis* (NMS G.2023.7.1)
Part C. Osteohistology

Part A. Summary and taxonomic status of parviraptorid specimens.

The squamate genus *Parviraptor* was first reported in a series of papers by Evans[8,13,67], based on specimens from five rock units spanning Middle Jurassic–Early Cretaceous. Caldwell et al.[7] reviewed this material and erected two new species and three new genera, in effect transferring the genus *Parviraptor* as used by Evans[8] to a higher-level group they called 'parviraptorids' (although this was not formally defined). Importantly, Caldwell et al.[7] stated that the *Parviraptor* hypodigm of Evans[8] was a chimaeric association of parviraptorid stem snakes and non-snake squamates belonging to other groups[7], with potential significance for understanding snake origins. Here, we discuss these arguments in context of new data from *Breugnathair elgolensis* (NMS G.2023.7.1), and other specimens. We do not attempt to address all aspects of the taxonomy here, which requires more detailed anatomical description of many specimens. For example, we provisionally accept the generic status of *Diablophis* and *Portugalophis*, pending discovery or description of more comprehensive material.

(1) Early Cretaceous Purbeck Limestone Group. Evans[8] reported two associations of squamate bones from the Early Cretaceous Purbeck Limestone Group of Dorset, UK: (1) NHMUK PV OR 48388, a group of skull bones in matrix from Durlston Bay, Dorset, which is the holotype of *Parviraptor estesi* Evans, 1994[8] (the type species of *Parviraptor* Evans, 1994[8]); and (2) NHMUK PV R8511, a group of skull bones, ribs and vertebrae from Swanage, Dorset, which she also referred to *Parviraptor estesi*. We provide CT scans and 3D meshes of these specimens in the current work, allowing detailed examination and comparisons (links in Supplementary Data 1).

NHMUK PV OR 48388. Caldwell et al.[7] regarded NHMUK PV OR 48388 as an association of bones from multiple different taxa, based on their criterion that bones referred to parviraptorids should be "snake-like". Of the elements present on NHMUK PV OR 48388, they retained only the left maxilla within *Parviraptor estesi* (as the holotype), stating that it was "clearly that of a snake" (Caldwell et al.[7], supplementary materials). Based on the absence of clear snake-like features, they removed the pterygoid, parietals and palatine of NHMUK PV OR 48388 from *Parviraptor estesi* and referred them to cf. Squamata indet. However, CT scans of NHMUK PV OR 48388 show that all these bones have substantial similarities to the

corresponding bones of NMS G.2023.7.1 (Extended Data Fig. 2), indicating that they do belong to parviraptorids. For example, the palatine of NHMUK PV OR 48388 was interpreted as a left palatine exposed in dorsal view by Evans[8], but as a right palatine exposed in ventral view by Caldwell et al.[7]. Our CT scans of NHMUK PV OR 48388 confidently confirm that this is a parviraptorid left palatine exposed in dorsal view. The strongest evidence for this comes from the presence of teeth on the surface embedded in the matrix (Extended Data Fig. 2), which are highly similar to the palatine teeth of NMS G.2023.7.1 (Fig. 3D) and resemble small versions of the maxillary teeth. As in NMS G.2023.7.1, the choanal margin of the palatine in NHMUK PV OR 48388 does not bear a choanal groove (Extended Data Fig. 2G).

There are several overlaps between the preserved anatomy of NHMUK PV OR 48388, and that of NMS G.2023.7.1. Both have parietals that share many features, consistent with referral to a single group (Fig. 3C, Extended Data Fig. 2D–E): they are paired and unsculptured, with a parietal foramen enclosed between the right and left elements; they are dorsoventrally shallow with shallow lateral margins and minimal development of ventral crests; they are significantly longer than they are wide; they have a long, flattened supratemporal process of similar length to the main body of the bone; the two parietals come together in the posterior midline to form a single or slightly bifid median process; and the ventral surfaces have a distinct longitudinal groove for the taenia marginalis, flanked medially by a low ridge. Nonetheless, there are differences in detail: parietals of NHMUK PV OR 48388 (Extended Data Fig. 2) are proportionally wider in the mid-section (ratio of anterior width to minimum width 1.48 vs 1.84 in NMS G.2023.7.1), lack a distinct nuchal shelf, and bear a deep ventral concavity between the base of the postparietal process and the base of the supratemporal process. If the slender element adjacent to the left parietal of NHMUK PV OR 48388 is a squamosal, as suggested by Evans[8], then they also differ by the narrower proportions of the squamosal of NHMUK PV OR 48388, and its lack of a distinct downturned tip.

Pterygoids are also preserved in NHMUK PV OR 48388 and NMS G.2023.7.1 and differ in which parts are preserved (Fig. 3A–B, Extended Data Figs 2I–K,7H–I). Notably, the palatal process of the pterygoid of NHMUK PV OR 48388, which is tooth-bearing in NMS G.2023.7.1, is missing. The preserved portions of these pterygoids show no notable differences other than those that may be attributable to

different preservation (the pterygoid of NMS G.2023.7.1 is broken into several parts that were digitally rearticulated, with some uncertainties).

NHMUK PV R8511. Caldwell et al.[7] also regarded NHMUK PV R8511 as a multi-taxon assemblage and identified only the frontal and vertebrae of that specimen as belonging to a parviraptorid, as aff. *Parviraptor estesi*. They labelled the frontal of NHMUK PV R8511 as a holotype, without further explanation (Caldwell et al.[7], supplementary figure 3), and removed other cranial elements and ribs on the NHMUK R8851 block from aff. *Parviraptor estesi*, referring them instead to cf. Squamata indet. (including the palatine and postfrontal/postorbitofrontal) or to 'miscellaneous indet.' (including the parietal and ribs). Caldwell et al.[7] specifically questioned whether the parietal belonged to a squamate, or was even a parietal, and they noted that it differed from the parietal of NHMUK PV OR 48388.

The frontal of NHMUK PV R8511 (Extended Data Fig. 3E–I) has a deep subolfactory process that appears notched on its posterior margin. Caldwell et al.[7] interpreted this notch as the anterior margin of an optic nerve foramen, thereby interpreting the specimen as having a snake-like suboptic shelf. However, the CT scans of the specimen show that the supposed notch has irregular margins and is due to breakage (Extended Data Fig. 3E) there is no corresponding lamina on the parietal (Extended Data Fig. 3A–C).

As with NHMUK PV OR 48388, all squamate bones on the NHMUK PV R8511 block are substantially similar to the corresponding bones of NMS G.2023.7.1 (Extended Data Fig. 3), providing no justification for excluding them from consideration as the bones of parviraptorids. There is also no secondary evidence that the squamate bones preserved on this specimen represent a mixed assemblage. It presents a set of bones of consistent size and consistent vertebral morphology, similar to other associated specimens from the Purbeck Limestone Group. The parietals of NHMUK PV R8851 and NHMUK PV OR 48388 show some differences that may suggest they belong to different species of parviraptorid, although we note that parietal shape can change quite markedly with ontogeny in squamates (e.g.[30]). Nonetheless both are similar overall to the parietals of NMS G.2023.7.1. The palatine of NHMUK R8851 is crushed onto the parietal and was originally identified by Evans[8] as a left palatine exposed in ventral view, and by Caldwell et al.[7] as a right palatine exposed in ventral view. CT scans of NHMUK PV R8511 indicate this is a right palatine exposed in dorsal view. The best evidence for

this is that a double row of teeth is present, on the downward-facing surface of this element (Extended Data Fig. 3), similar to the condition in both NHMUK PV OR 48388 and NMS G.2023.7.1. These teeth are only visible in our CT scans with difficulty (Extended Data Fig. 3D; due to the relatively larger size of the NHMUK R8851 block, the quality of our CT scans is low), and their detailed morphology cannot currently be determined.

There are few overlaps between the preserved anatomy of NHMUK R8511, and that of NMS G.2023.7.1. However, both have parietals with overall similar morphology, including the presence of a pronounced nuchal shelf (Extended Data Figs 2D–E 3A–C). The parietal of NHMUK R8511 is proportionally longer in relation to its width than that of NMS G.2023.7.1, and has a more pronounced postparietal process, flanked by a ventral concavity (like that of *P. estesi*, but unlike NMS G.2023.7.1). The posterior emargination between postparietal and supratemporal processes is strongly 'U' shaped, in contrast to the more angular shape in NMS G.2023.7.1.

Given the evidence presented here that the squamate bones on both Purbeck blocks all belong to parviraptorids (contra Caldwell et al.[7], who believed that they belonged to multiple distinct groups of squamates), there is no particular reason to believe that these sets of elements each represent more than one individual. We therefore treat each specimen (NHMUK PV OR 48388 and NHMUK R8511) as a single OTU in our analyses. As their specific distinction is uncertain, we conservatively score them as separate tips in our phylogenetic analyses.

Comments on phylogenetic scores for maxilla, based on the holotype of Parviraptor estesi *(NHMUK PV OR 48388).* Caldwell et al.[7] presented a series of phylogenetic scores for the matrix of Gauthier et al.[27]. The resulting analysis supported stem snake affinities for parviraptorids, similar to the results of some of our analyses. The phylogenetic scores *Parviraptor estesi*, used in the present study differ in places and are explained here.

Character 5 of ref.[27]. Premaxillary-maxillary fenestra: (0) absent; (1) present. Caldwell et al.[7] scored state 0 in *Parviraptor* and *Portugalophis*. State 0 is widespread among snakes and non-snake squamates. However, we were cautious about scoring this character given that premaxillae are not preserved. We therefore scored it as '?' in the versions of this character used in our analyses.

Character 9 Premaxilla-maxilla suture: (0) firm; (1) loose. Caldwell et al.[7] scored state 1 in *Parviraptor* (and '?' in other parviraptorids). State 1 is also present in snakes, mosasauroids and rhynchocephalians. We were cautious about scoring this character given the absence of a preserved premaxilla, and therefore scored it as '?' in the versions of this character in our analyses. We note the presence of a small notch on the premaxillary process of the maxilla in NHMUK PV OR 48388, which raises the possibility of an interlocking suture, despite the narrow and rounded morphology of this process. Nevertheless, the state of this character remains uncertain.

Character 20 of ref[27]. Nasal-maxilla suture: (0) present; (1) absent. Caldwell et al.[7] scored state 1 in *Parviraptor* and *Portugalophis*, and this is shared with some anguimorphs, plus mosasauroids and snakes. However, we were cautious due to the absence of a complete frontal and absence of preserved nasals, and therefore scored this as '?' in all our parviraptorids, in all versions of this character used in our analyses.

Character 119 of ref.[27]. Maxilla firmly sutured to palatine: (0) present; (1) prominent palatine process of maxilla; (2) loosely ligamentous connection via projecting palatine process of maxilla and distinct maxillary process of palatine, with the former lying anterior to the latter; (3) maxilla free of palatine, suspended from prefrontal; (4) maxilla rotates to erect fang. Caldwell et al.[7] scored state 1 in *Portugalophis* (1) (and '?' in other parviraptorids), which is shared with snakes. However, *Breugnathair* and *Parviraptor* both show clear lateral articular surfaces on the palatine for contact with the maxilla. We are therefore confident in scoring state 0 in these taxa, in versions of this character used in our analyses.

Character 135 of ref.[27]. Prefrontal-maxilla articulation: (0) prefrontal posteroventromedial corner narrowly (or not at all) in contact with maxilla lateral to palatine; (1) prefrontal broadly contacts maxilla supradental shelf lateral to palatine; (2) prefrontal has mobile contact with maxilla; (3) rod-like prefrontal arched dorsally, bifid at each end, with mobile joints at maxilla and frontal (prefrontal functionally part of upper jaw). Caldwell et al.[7] scored state 2 in *Parviraptor*, *Portugalophis* and *Diablophis*. State 2 is uniquely shared with some snakes. However, the palatine of *Breugnathair* and *Parviraptor* bears a dorsal articular facet for contact with the prefrontal. Therefore, although the facial process of the maxilla is low in *Parviraptor* (and *Portugalophis*), there was nevertheless likely a stabilizing contact resulting in a

non-mobile prefrontal-maxilla articulation. We therefore scored this as state 0 in versions of this character used in our analyses.

Character 423 of ref.[27]. Position of marginal teeth relative to tooth-bearing element: (0) on medial side of tooth-bearing element; (1) near/on apical margin of tooth-bearing element. Caldwell et al.[7] scored state 1 in *Parviraptor*, *Portugalophis* and *Diablophis*, which is shared with snakes, some mosasauroids, and acrodont taxa. However, under the definitions of Gauthier et al.[27], these taxa would not be considered to have apically set teeth. The definitions of character states used in the datasets we analysed vary slightly, with Dataset 3[47] having only two states ('medial' or 'apical'), and the others[3,6] distinguishing between those states and 'apicolingual' (or equivalent), which is correct for parviraptorids. We therefore scored this state 2 ('apicolingual') in Dataset 1[3], state 1 (medial but within the upper 20% of dentary height) in Dataset 2[6], and state 0 ('medial', also shared with the stem snakes *Coniophis* and *Najash*) in Dataset 3[47].

(2) Middle Jurassic Forest Marble Formation. Evans[8] referred portions of dentaries, a partial maxilla, frontals, parietals, and vertebrae obtained via screenwashing of bulk sediments from the Middle Jurassic Forest of Kirtlington, Oxfordshire, UK to *Parviraptor* cf. *estesi*, signalling that the specimens share features with *P. estesi* but cannot be referred to that species (i.e. that these bones belong to parviraptorids, as used here). These specimens are from the Bathonian stage, and are therefore penecontemporaneous with NMS G.2023.7.1.

Caldwell et al.[7] argued that the Kirtlington bone assemblage represented multiple distinct taxa and mostly did not belong to parviraptorids. They erected a new genus and species of parviraptorids, *Eophis underwoodi*, based on NHMUK PV R12355 (as holotype), R12354 and R12370 (shown here, in Extended Data Fig. 4A–B). The text of Caldwell et al.[7] assigns NHMUK PV R12354 and R12370 as 'syntypes' whereas the figure captions assign NHMUK PV R12355 as the holotype and other specimens as paratypes. We provide CT scans and 3D meshes of these specimens in the current work, allowing detailed examination and comparisons (links in Supplementary Data 1). We specifically compare the overlapping cranial elements shared with NMS G.2023.7.1: a parietal and dentary portions.

NHMUK PV R12353 is an isolated left parietal from Kirtlington (Extended Data Fig. 4H–K). It is smaller than that of NMS G.2023.7.1 and the supratemporal process

has broken off. Nonetheless, it shares the same general morphology as the specimens from Skye and Purbeck. Similar to NMS G.2023.7.1 and NHMUK PV R8851, there is a nuchal shelf that extends across the full posterior width of the bone, although this appears somewhat deeper in NHMUK PV R12353. As in the Purbeck parietals, the dorsolateral edge of the bone is sharp rather than rounded, and the anterior part of the bone is more flared.

The three parviraptorid right dentary portions from Kirtlington[7,8] likely come from different individuals given that they were obtained by screenwashing. They include a symphysial portion bearing five alveoli (NHMUK PV R12355; the holotype of *Eophis underwoodi* Caldwell et al., 2015[7]), a central portion bearing six alveoli (NHMUK PV R12354), and a posterior portion bearing three alveoli and an intradental lamina (NHMUK PV R12370) (Extended Data Fig. 4A–C). These specimens are smaller than NMS G.2023.7.1. For example, the height of the posterior part of NHMUK PV R12355 is 1.35 mm, compared to 1.8 mm for the equivalent measurement in NMS G.2023.7.1.

The dentary portions from Kirtlington (Extended Data Fig. 4A–C) share many features with the dentary of NMS G.2023.7.1, supporting their attribution to parviraptorids. Differences between NMS G.2023.7.1 and the Kirtlington dentary fragments may reflect either taxonomic or ontogenetic variation: (1) the interdental plates are well-defined, high triangular plates in the Kirtlington specimens, but are lower, and less well-defined in NMS G.2023.7.1, especially mesially and distally. We note that the development of interdental ridges and plates can vary ontogenetically in squamates[68]. (2) The dentary symphysis is much more prominent in NMS G.2023.7.1, extending posteriorly as a distinct posterodorsal flange that is absent in NHMUK PV R12355. This difference may reflect ontogenetic differences in the degree of ossification of the symphysis. (3) The subdental lamina of NHMUK PV R12354, the Kirtlington mid-section, is proportionally low (around one-sixth of dentary height in NHMUK PV R12354) compared to that of NMS G.2023.7.1. However, we also note that the subdental lamina of the holotype symphysial portion of the dentary of *E. underwoodi* is proportionally similar to that of NMS G.2023.7.1 (see below).

The taxonomic status of *Eophis underwoodi* is important for the current work because it is from similar-aged (Bathonian, Middle Jurassic) sediments to NMS G.2023.7.1. The diagnosis of *E. underwoodi* refers to the small size of the

specimens, and depth of the subdental lamina as distinguishing it from other parviraptorid species: “Small-bodied snake with low and shallow subdental lamina of dentary. Differs from both *Diablophis gilmorei* and *Portugalophis lignites* in having smaller subdental lamina relative to dentary size” (Caldwell et al.[7], p.5). However, size may not usefully distinguish between species given that individuals grow larger through ontogeny, and especially given the evidence of prolonged growth duration in parviraptorids (e.g. growth continued over at least nine years in *Breugnathair*, see main text osteohistology; Fig. 2). Further evidence that size differences may not be useful for parviraptorid taxonomy comes from ontogenetic size variation within the Kirtlington assemblage of parviraptorid bones (e.g. vertebrae[8]). This leaves only the relatively shallower subdental lamina as a distinguishing feature. However, although this is present in the NHMUK PV 12354 (Extended Data Fig. 4A–C; dentary mid-section), it does not seem to be especially narrow in the holotype of *E. underwoodi* (NHMUK PV R12355). The ratio of the subdental lamina height below the fourth alveolus, to the straight line distance from the dorsomesiolingual point of the symphysis, to the midpoint of the posterior margin of the fourth alveolus is 0.29 in *E. underwoodi* (NHMUK PV R12355), 0.30 in *Breugnathair*, and 0.23 in *Portugalophis*. The ratio of the subdental lamina height below the fourth alveolus, to the straight height of the lateral surface of the dentary at the level of the fourth alveolus is 0.38 in *E. underwoodi* (NHMUK PV R12355), 0.35 in *Breugnathair*, and 0.36 in *Portugalophis.* The differences between these values are small, and a robust diagnosis is not possible based on such incomplete material. We also cannot exclude that metric differences are possibly linked to small size or immaturity. We therefore regard *Eophis underwoodi* as a nomen dubium and Parviraptoridae indet. Unfortunately, we also understand (pers. comm. Marc E. H. Jones, Natural History Museum, 2024) that NHMUK PV R12355 is no longer in its original container and cannot be traced.

(3) Late Jurassic Guimarota locality. Evans[8] referred specimens from the Late Jurassic Guimarota locality of Portugal to *Parviraptor* cf. *estesi*. Caldwell et al.[7] specified a left maxilla (MG-LNEG [Museu Geologico, Lisboa, Portugal] 28091) from this assemblage as the holotype of a new genus and species of parviraptorid, *Portugalophis lignites*, including a left dentary (MG-LNEG 28091) as a paratype, and a partial left maxilla (MG-LNEG 28100) as a referred specimen.

Material of *Portugalophis* is larger than that of other parviraptorids[7], but the taxonomic usefulness of size differences is difficult to evaluate in a reptile with protracted growth. Nevertheless, the dentary of *Portugalophis* differs from that of NMS G.2023.7.1 by having taller interdental ridges, less sharply recurved teeth (40° posterodorsally vs 50° in NMS G.2023.7.1), dorsoventrally deeper proportions, and a different morphology of the splenial facet which, though incomplete in *Portugalophis*, forms a narrow gradually widening groove along the ventral margin of a deep subdental ridge and lacks a ventral lamina for the prearticular facet. In NMS G.2023.7.1, the splenial facet extends obliquely (posterodorsally) across the subdental ridge to a greater degree, so that the ridge narrows more markedly, and gives rise to a ventral lamina (separate from and medial to, the intramandibular septum) that may have supported the anterior part of the prearticular.

Portugalophis was not included in our phylogenetic analysis because it is relatively incomplete and generally duplicates information already represented by the Skye and Purbeck specimens. Nonetheless, we hope that the descriptions and illustrations of parviraptorid bones from other localities, provided here, will facilitate identification of further cranial and postcranial elements from Portuguese localities.

(4) Middle Jurassic Kilmaluag Formation. Evans & Waldman[67] reported *Parviraptor*-like vertebrae from the Middle Jurassic Kilmaluag Formation of Scotland. These were not reported with specimen numbers and have not been discussed further in subsequent literature and could not be relocated during the current work.

(5) Late Jurassic Morrison Formation. Evans[13] reported LACM (Los Angeles County Museum, Los Angeles, USA) 4684/140572, an association of bones from the Late Jurassic Morrison Formation of Fruita Paleontological Area, Colorado, as the holotype of a new species *Parviraptor gilmorei* Evans, 1996. Caldwell et al.[7] made this the specimen type species of a new genus of parviraptorids, *Diablophis gilmorei*, restricting the holotype to the maxilla, dentary and axis vertebra.

The right dentary of *Diablophis gilmorei* is preserved[7,8] (LACM 4684/140572) and differs from NMS G.2023.7.1 in several conspicuous ways, although the specimens are similar in size. Most notably: (1) The subdental gutter is proportionally wider in LACM 4684/140572, approximately 1.3 times the width of the alveolar row. (2) The crowns of LACM 4684/140572 have a swollen basal portion and have apices

that are not sharply recurved compared to NMS G.2023.7.1 or other parviraptorids. (3) The alveoli and tooth bases have anteroposteriorly elongate oval cross-sections (length:width ratio of mid-series alveoli = 1.5 compared to 1.3 in NMS G.2023.7.1).

Material of *Diablophis gilmorei* was not studied further during the present work, and was therefore not included in our phylogenetic analyses, pending further description.

Part B. Summary description of *Breugnathair elgolensis* (NMS G.2023.7.1)

Elements represented. Cranium: left jugal, left postfrontal, left and right parietals, left and right squamosal, braincase, left vomer, left palatine, right pterygoid. Mandible: right dentary, right angular, right compound element incorporating surangular, prearticular and articular. Axial skeleton: 32 vertebrae or partial vertebrae, many dorsal ribs, a cervical intercentrum. Appendicular skeleton: right coracoid, right and left humeri missing distal ends, right ilium, fragment of right pubis, right femur missing distal end, left femur, missing epiphyses, right tibia, missing epiphyses, possible fibula, one metapodial, three phalanges, including one ungual phalanx.

Skull

Parietal. Both parietals are preserved (Figs 3C, Extended Data Fig. 6B–C), of which the left is more complete. The shared size, morphology, and detailed fit of their midline articulation shows they belong to a single individual. The left parietal preserves its posterolateral supratemporal ramus in several pieces and parts of its anterior articulation for the frontal.

The parietals have a flat, unsculptured dorsal surface and are proportionally long, with a ratio of anteroposterior length to minimum transverse width of 4.0. They are narrowest just anterior to the nuchal fossa. Dorsal and ventral facets along the anterior margin suggest that the frontal and parietal fitted closely into one another with little possibility of mesokinetic flexion.

The lateral surface of the left parietal bears a short, anteroposteriorly oriented facet for articulation with the postfrontal. Posterior to this facet, the parietal margin is shallow and vertical, with the adductor muscles neither invading the dorsal surface nor attaching to the underside of a lateral shelf.

The medial border of the parietal is notched for a small parietal foramen at around one-half of the parietal length. This margin thickens posteriorly and is excavated by a linear ventral recess (parietal fossa), open posteriorly, that accommodated the processus ascendens of the supraoccipital. The ventral surface of the parietal bears a narrow longitudinal groove for the taenia marginalis of the chondrocranium, flanked by a broad ridge that extends posteriorly into a short median postparietal (=posteromedian) process. A long supratemporal process, approximately equal in length to that of the main body of the parietal, extends posterolaterally at about 45 degrees. On its lateral surface, it bears a deep slot facet, for either the squamosal or the supratemporal.

Between the postparietal and supratemporal processes, the posterodorsal surface of the parietal bears a wide U-shaped nuchal fossa that extends on to and occupies the entire dorsal surface of the supratemporal process. The two postparietal processes come together in the midline to form a single median process that separates the right and left nuchal fossae across the midline.

Jugal. The preserved left jugal (Extended Data Fig. 6D–F) is missing the dorsal (postorbital) process and the anterior end of the maxillary process. The posteroventral corner is rounded, with no development of a posterior spur or tuberosity. The orbital margin of the jugal is thickened and forms a robust, medially-projecting ledge. A ridge overhangs the maxillary facet, so that, at least posteriorly, the jugal overhung the maxilla at their articulation.

Postfrontal. The left postfrontal (Fig. 3A, Extended Data Fig. 7A) is triradiate in dorsolateral view, with a long anterior (frontal) process, a short tab-like posterodorsal process (part of which is missing), and a broken ventrolateral (postorbital) process that is triangular in cross-section. The posterior margin of the postfrontal is smooth (unfaceted) and formed part of the anterior margin of the upper temporal fenestra. The ventral end of the postorbital process, including the postorbital facet, is missing.

Postorbital. No postorbital bone has been identified. However, given the length and orientation of the supratemporal process of the parietal, the long slender squamosal must have been braced anteriorly by a postorbital component. This is confirmed by the long anteromedial facet on the squamosal itself (see below).

Squamosal. Both squamosals are preserved and the right is more complete (Fig. 3A, Extended Data Figs 6A,7E). It is a long, band-like bone that curves posterolaterally towards its contact with the quadrate. The anterior part is mediolaterally compressed and becomes more triangular in cross-section posteriorly. The medial surface bears a long postorbital facet that is shallow anteriorly and deepens posteriorly. A down-curved posterior tip met the quadrate.

Vomer. The right vomer is preserved (Fig. 3B, Extended Data Fig. 7F–G). Its elongate proportions reflect the long maxilla and, apparently, extended rostrum of other parviraptorids[8] (based on NHMUK PV OR 48388). The vomer is blunt anteriorly where it likely abutted the premaxilla (based on the presence of a low, vertical anterior surface). The lateral margin is emarginated between the premaxillary process and choanal margin, creating a notch for the opening of the vomeronasal organ.

The ventral (palatal) surface of the vomer is smooth (no midline or lateral crest). It is perforated by a large anteromedial foramen that opens into a groove that extends forward on to the premaxillary process. A single, oblique row of small, recurved teeth extends posteromedially from approximately the mid-length of the vomer.

The dorsal surface of the vomer is weakly concave (mediolaterally) but is otherwise relatively featureless, except for the large anterior perforating foramen and a tongue-shaped posterior facet for the vomerine process of the palatine. The medial margin of the vomer is straight and weakly facetted for contact with the contralateral element anteriorly only.

Palatine. The left palatine is preserved, including the central portion and the lateral (maxillary) process (Fig. 3B,D). The posterior (pterygoid) and anterior (vomerine) processes are incomplete, as is the thin, medial part of the palatine. Two rows of recurved palatine teeth are present and are larger than those of the vomer but smaller than those of the dentary. A shallow transverse concavity on the dorsal surface of the palatine anteriorly likely contacted the descending flange of the prefrontal, but the lack of a strong rugosity here suggests the two bones were not strongly sutured.

The lateral maxillary process of the palatine is robust, with a narrow neck and an anteroposteriorly expanded 'foot' that contacted the maxilla. Seen in lateral view, the 'foot' is divided into a long ventral component bearing a slot-like maxillary facet and a shorter flange, apparently incomplete, that may also have abutted the maxilla. Between these two components is a gap that would have carried the maxillary nerve and accompanying blood vessels into the body of the maxilla.

Pterygoid. The left pterygoid is preserved in three parts (Fig. 3A–B, Extended Data Fig. 7H–I), missing only part of the midsection connecting the palatal plate to the quadrate process, and a small part of the pterygoid flange. The palatine process likely bordered a wide interpterygoid vacuity (based on comparison of the pterygoid to the braincase width; Fig 3B). The palatine process is not bifurcated and bears both dorsal and ventral facets at its anterior end, suggesting that it slotted firmly into the palatine. The pterygoid bears a row of small, recurved teeth along the medial margin of the palatine process. The row is single anteriorly but becomes double posteriorly as a short row of smaller teeth is added medial to the main row. The pterygoid flange is directed anterolaterally and has a concave anterior margin that formed the posterior border of the suborbital fenestra. The tip of the flange is broken but what remains bears an anterodorsomedial facet for the medial head of the ectopterygoid.

The quadrate process of the pterygoid forms a thick mediolaterally compressed strut of bone with a sharp dorsal edge and a rounded ventral one. The anterior two-thirds of the quadrate process are relatively straight, but the posterior end curves laterally to meet the quadrate. The anterodorsolateral end of the process bears a deep fossa columellae for the epipterygoid. The medial surface of the quadrate process bears a shallow ovoid fossa for a synovial contact with the basipterygoid process of the basisphenoid.

Braincase. The braincase of NMS G.2023.7.1 is relatively complete (Fig. 3B, 4B–C, Extended Data Fig. 7A–C), although it is dorsoventrally compressed. It is missing the end of the right paroccipital process and the basisphenoid, and has damage to the medial walls of the otic capsules and anterior parts of the prootics. Sutures between the components are visible, but are well-developed and tight-fitting, confirming the maturity of the skeleton. Overall, the endocranium of NMS G.2023.7.1 is similar to

those of non-snake squamates, and lacks numerous snake-like features, notably the development of the crista circumfenestralis creating a juxtastapedial recess around the fenestra vestibuli and lateral opening of the recessus scalae tympani (LRST). The specimen also possesses numerous features that are lost in snakes, including the processus ascendens, post-temporal fenestrae and the metakinetic axis, free-ending crista alaris of the prootic, and a large paroccipital process[28,36]. All of these snake features, except the development of the crista circumfenestralis, are present in the early diverging limbed snake *Najash*[24], which also differs from NMS G.2023.7.1 in having a massive fenestra vestibuli filled by a large stapedial footplate, a feature found also in the primitive snake *Dinilysia* and in madsoiids, and usually indicative of a fossorial lifestyle.

The **supraoccipital** is a broad, relatively shallow bone that is almost pentagonal in shape, with posterolateral wings roofing the vestibular cavity, anterolateral wings articulating with the prootics, and a short ossified median base to the processus ascendens (Extended Data Fig. 7B). There is no sagittal crest. Posteriorly the supraoccipital contribution to the dorsal margin of the foramen magnum is limited by the expanded otoccipitals.

The left **oto-occipital** is better preserved than the right (Fig. 4B–C, Extended Data Fig. 7A). The paroccipital process is short and wing-like, with a deep distal edge that inclined slightly anterodorsally and is very thin. The body of the oto-occipital (opisthotic+exoccipital) is almost horizontal where it meets the supraoccipital. The exoccipitals are fully fused to the opisthotics, forming the posterior margins of the vagus foramina dorsally and of the lateral opening of the recessus scalae tympani (LRST) ventrally. A shallow ventrolateral wing of the exoccipital component excludes the basioccipital from the lateral margin of the LRST. Medially, however, the basioccipital enters the margin of the medial opening of the recessus scalae tympani. Externally, the body of the exoccipital is perforated by two foramina for the hypoglossal nerve (XII), and these are separate from the vagus canal (X). Posteromedially, each exoccipital contributes to the lateral corners of the occipital condyle. As seen in lateral view (Fig. 4C), the large anterior surface of the paroccipital wing overhangs the fenestra vestibuli, but the margins of this fenestra are broken on both sides so neither its orientation nor relative size can be estimated. A crista interfenestralis separates the fenestra vestibuli from the LRST.

The left **prootic** is better preserved than the right (Fig. 4C, Extended Data Fig. 7A). Posteroventrally the prootic contributes to the margin of the fenestra vestibuli, and posterodorsally it forms a wing that braces the anterior edge of the paroccipital process. The prootic extends beyond its contact with the supraoccipital as a moderately developed alary process. The tip of each alary process is blunt-ended and may have been continued in cartilage, but the small cross-section of the terminal surface suggests it did not extend much further.

A low, rounded horizontal ridge on the lateral surface of the prootic marks the path of the lateral semi-circular canal. In its posterior one third, this low ridge is extended by a relatively weak crista prootica that ends just dorsal to the fenestra vestibuli. Anteroventral breakage precludes description of the path of the facial nerve canal (VII), or determination of the original shape and depth of the incisura prootica, and the presence or absence of an anterior inferior process. However, there is no supratrigeminal process and clearly no contact between the alary process and the parietal.

The **basioccipital** is trefoil-shaped, with a large, projecting posterior occipital condyle and extended horizontal posterolateral wings that articulated with the crista tuberalis of the exoccipital and floored the recessus scalae tympani (Fig. 4B, Extended Data Fig. 7A). The occipital condyle is U-shaped as seen in posterior view, but an irregular groove on its the posterior surface is the impression of a vertebra that was preserved crushed into the occipital condyle.

Dentary. The right dentary preserved, missing its posterior and posteroventral parts (Fig. 3A, Extended Data Fig. 7J–L). The preserved portion of the dentary is 19.5 mm long and bears 19 alveoli, although more may have been present posteriorly before breakage. Small amounts of alveolar bone are present, forming low interdental septa that expand lingually into low, triangular interdental (lingual) plates that define the boundaries of individual alveoli. Channels from the alveolar canal (carrying nerves and blood vessels) open into the alveoli at their distal margins. The labial wall of each alveolus (pleura) is low so that the lingual and labial margins of the tooth base are almost symmetrical, with the pulp opening directed ventrally not labially. This arrangement differs from the pleurodont implantation (sensu refs[37,38]) of stem-lepidosaurs, the Cretaceous stem squamate *Oculudentavis*, most non-snake squamates, and early-diverging rhynchocephalians where there is a stronger labial

pleura and asymmetric teeth. It also differs from the acrodont implantation (sensu refs[37,38]) of most rhynchocephalians, acrodontan lizards and trogonophid amphisbaenians. The condition in *Breugnathair* and other parviraptorids is closer to that in many snakes, where the tooth base sits in a shallow alveolus[37], is symmetrical, and has the pulp cavity opening ventrally[39]. The shape of the alveolus differs (more medio-distally compressed in snakes) and snakes lack the subdental gutter, so that the alveolus extends across the full width of the jaw[38].

The dentary curves anteromedially at its mesial end, terminating in an expanded symphysial surface. The Meckelian fossa opens just ventral to the symphysial surface, incising its ventral margin. The lateral surface of the dentary is convex, with a pronounced ventromedial curvature. It bears a row of least five nutrient foramina just dorsal to mid-height, the posteriormost of which is located at the level of the tenth alveolus. A shallow muscle scar (genioglossus or intermandibularis) occupies the ventrolateral surface of the dentary ventral to these alveoli.

A cross-section of the dentary at its widest preserved portion has a mediolateral width (2.4 mm) only slightly less than its dorsoventral height (2.8 mm). These mediolaterally broad proportions result from the presence of a wide subdental gutter ventrolingual to the alveoli, that is approximately as wide mediolaterally as the alveolar row. The subdental lamina is dorsoventrally deep, constituting up to one-third of the dorsoventral height of the dentary and extending dorsally as a low anteroposteriorly oriented crest that bounds the subdental gutter medially. The Meckelian fossa is not enclosed or occluded at any point. It opens ventrally at the anterior end of the dentary, where it is mediolaterally narrow, and becomes progressively broader distally.

Splenial. The splenial has not been identified on the NMS G.2023.7.1 block, but a facet along the dorsal margin of the Meckelian fossa in the posterior one third of the right dentary demonstrates its presence as an element separate from the dentary (Extended Data Fig. 7J).

Angular. The right angular is small relative to the other jaw elements (Fig. 3A, Extended Data Fig. 7J–L). It is triangular in cross-section with the three faces of the bone forming medial, ventral and dorsal surfaces. The dorsal surface is perforated at

its midpoint by a large foramen for a branch of the mandibular nerve that opens on to the medial surface of the bone at the posterior mylohyoid foramen.

The anterior tip of the angular tapers anterodorsally and was probably clasped by the splenial, or between the splenial and dentary. In ventral view, the angular is divided into a thin lateral lamina and a rugose medial ridge. This ridge ends abruptly at the junction of the anterior and middle thirds of the bone where a ventromedial groove crosses the rugose ridge obliquely from medial to lateral. The combination of the grooved surface and tapering tip suggests the joint between the angular, splenial and dentary was complex, with the angular forming a superficial socket for the splenial, but also bracing it laterally (within the mandible), although this is difficult to reconstruct without the splenial itself or the posteroventral margin of the dentary. It was not an abutting joint, nor an obviously rotatory one, given the tapering angular process bracing the splenial medially. The condition in NMS G.2023.7.1 seems to be most closely similar to that in the extant varanoid *Lanthanotus*, in which the posteroventral end of the splenial abuts a ventromedially positioned notch in the angular as visible externally[36] but overlaps the splenial internally as in many other non-snake and non-mosasaurian squamates[27].

Compound bone. The **surangular, prearticular and articular** of the right mandible are fused into a single compound element that is 21.9 mm long from its anterior preserved end up to the posterior lip of the articular cotyle (Fig. 3A, Fig. 7K–L). This element is deepest at the level of the anterior surangular foramen. The anterodorsal surface of the surangular bears a large coronoid facet. The posterior half of the compound bone forms a shallow dorsomedially-facing adductor fossa, bordered dorsally by the surangular and ventrally by the prearticular.

The posterior end of the compound bone is robust and bears a short deep, transverse articular fossa for the quadrate and a short rounded retroarticular process oriented at roughly 45 degrees to the horizontal. Caldwell et al.[7] provisionally referred an isolated surangular bone to *Diablophis*. If correctly attributed, it might suggest different levels of mandibular co-ossification existed in parviraptorids or be a reflection of ontogenetic stage.

Dentition

Eleven erupted teeth are present in situ and many replacement crowns are present within alveoli or in the subdental gutter (Extended Data Fig. 7J–L). More anterior replacement teeth are located essentially medial to the erupted tooth base whereas more posterior replacement teeth are located distolingually. Medial to the 11th alveolus, there are two tooth crowns, one posterior to the other (Extended Data Fig. 7J,L). The more anterior of these is larger than that following, suggesting these represent two replacement generations of the same alveolus and that tooth replacement was relatively rapid in NMS G.2023.7.1. Replacement teeth are all unattached and most lie horizontally as preserved, with their apices oriented anteriorly or posteriorly. However, a vertical replacement tooth is present medial to the fourth alveolus, and the varying orientations of other replacement teeth suggests at least some disarticulation has taken place. Therefore, it is uncertain whether replacement teeth developed vertically, as in most other squamates, or horizontally as in snakes[38].

Teeth are highly tapering, circular in cross-section (without carinae), and recurved such that their apices point posterodorsally at an angle of approximately 50° from vertical. Most of the implanted teeth show no evidence of basal erosion. Nevertheless, small embayments are present lingually in the bases of the fourth and ninth erupted teeth, and may be related to resorption during replacement, and the alveolar bone is recessed in places to accommodate the replacement teeth.

Postcranial skeleton

Vertebral column. Thirty-two complete or partial vertebrae are preserved on the NMS G.2023.7.1 block (Fig. 1C–I, Extended Data Fig. 6G–L), including the axis and at least five further cervicals (giving a minimum of seven cervicals counting the missing atlas), one cervicodorsal, 17 definite dorsals, three possible dorsals, three proximal caudals and two possible caudals or sacrals (Supplementary Data 2). There is also a free intercentrum, probably cervical due to the midventral ridge. The recovery of caudal hemi-vertebrae at Kirtlington suggested that parviraptorids had caudal autotomy[8], and this could explain the absence of more distal caudals. Individual vertebrae are referred to by an alphabetical code where ‘CE’ refers to cervicals (e.g. ‘CAa’, Cab’ etc), ‘D’ to dorsals, ‘CA’ to caudals, ‘S’ to sacrals, and uncertainties are indicated with hypens (e.g. ‘CE-D’ for the cervicodorsal)

(Extended Data Fig. 5; Supplementary Data 2). The original order of the vertebrae along the column is not known.

Twenty-four definite presacral vertebrae, plus three possible dorsals, are preserved giving a minimum presacral count of 25–28 once including the missing atlas. We use an estimated original presacral count of 27–30 for our body length reconstruction (Fig. 1A), but this should not be taken as firm anatomical knowledge (see *Methods*). All vertebrae are procoelous with a round, fully closed posterior condyle, although the condyles of several vertebrae, for which our CT scan data is especially clear, bear a trace of the notochordal pit at their centre (two cervicals [Cea, CEb] and two anterior caudals [CAa, CAb]). Notochordal pits may be present on other vertebrae too, but this is difficult to determine due to the varying quality of our CT data.

The condyles of presacral vertebrae (Fig. 1C–F, Extended Data Fig. 6G–L) are rounded and face posteriorly. Correspondingly, the cotyles face anteriorly rather than anteroventrally, and so are essentially hidden in ventral view[15].

Axis vertebra. The axis vertebra (Extended Data Fig. 6G–I) has a moderately tall neural spine set back over the posterior half of the axis body. There are fully developed pre- and post-zygapophyses, with the prezygapophyseal facets both prominent and convex, facing dorsolaterally. The dens is fused to the body of the axis without an obvious trace of a suture.

The ventral surface of the axial centrum bears a wide midline ridge that is approximately square in cross-section, and bears a shallow midline concavity anteriorly, just posterior to the hypapophysis. An intercentrum is fused in place ventrally. It has a pronounced midline ridge and short posterolateral processes that are directed more posteriorly than laterally, resulting a three-fold ventral structure that is also present in an axial centrum from Kirtlington (NHMUK R12360). Caldwell et al.[7] described this as a 'trefoil' hypapophysis and stated that it was also present in some gekkotans, referring the Kirtlington axis to cf. Gekkota taxon incertae sedis A. However, the axial intercentrum is also trefoil-shaped (apex anterior) in the dibamids *Anelytropsis* and *Dibamus*, as well as some scincids (e.g. *Acontias*, *Melanoseps*)[46], undermining the referral of NHMUK R12360 to Gekkota specifically, based on this feature.

There is no prominent posterior hypapophysis or raised structure for articulation with the third cervical intercentrum. This is similar to most squamates and their outgroups, but differs from the condition in mosasauroids, some anguimorphs, snakes, and a few other taxa, in which the third intercentrum is entirely or primarily on the posteroventral surface of the axial centrum[15,19,44,45].

A pronounced lateral crest extends posterodorsally along the axis. The anterior portion of this crest, at the level of the axial prezygapophysis, projects laterally, resembling a rib articulation. This lateral projection may have given attachment either to a rib, a muscle tendon, or a ligament.

Postaxis cervical vertebrae. Typical cervical vertebrae (Fig. 1C–F) have proportionally short centra (e.g. length:posterior condyle width ratio = 1.47 [CEb] or 1.38 [CEb]) that are almost triangular in ventral view, widening anteriorly due to the presence of wing-like rib synapophyses and a prominent posterior condyle. The condyle is narrower than the posterior margin of the centrum, creating a step in the outline seen in ventral view. The ventral surface of the centrum bears a prominent, sharp midventral crest along its entire anteroposterior length.

All of the preserved post-axis cervicals bear rib synapophyses. This suggests that one or more anterior cervicals may be missing, as limbed lizards typically have at least one, and up to three, ribless post-axis cervicals. However, there are a few extant gekkotan and scincid taxa in which the first cervical rib is on C3[15] and the axis bears a rib in *Dibamus* and *Anelytropsis*[46].

The zygapophyses are widely spaced with prezygapophyseal facets facing dorsomedially and postzygapophyseal facets facing ventrolaterally, both at approximately 45°. Prominent, dorsolaterally-facing zygantral articulations are present as continuations of the prezygapophyseal facets.

Cervical intercentrum. A single intercentrum is preserved, probably from the anterior cervical region as it resembles that associated with the axis. It comprises a shallow ‘U’ shaped horizontal bar that bears facets for articulation with centra on its dorsal surface, and a pronounced ventral keel. The morphology of the facets indicates that the intercentrum articulated with the pleurocentra anterior and posterior to it, as in rhynchocephalians, *Oculudentavis*, gekkotans and xantusiids[15,33]. This differs from the condition in scincids, dibamids, amphisbaenians,

mosasauroids, anguimorphs, and the early stem snake *Dinilysia* in which the intercentrum articulates primarily or entirely with the preceding centrum and may be fused with it[15], and in crown snakes where the hypapophyses are thought to be pleurocentrally derived rather than modified intercentra[27].

Dorsal vertebrae. Dorsal vertebrae (Extended Data Fig. 6J–L) have a proportionally long centrum (centrum lengths of sufficiently well-imaged dorsal vertebrae are 3.5–4.2 mm compared to 3.3–3.7 mm for cervical centra; all measurements excluding the condyle; Supplementary Data 2) and have condyles/cotyles that are slightly wider mediolaterally than deep dorsoventrally, differing from cervicals in which condyles/cotyles are deeper than wide. Most dorsals have broad, approximately flat (Db), or mediolaterally convex ventral surfaces (e.g. Df, Di), lacking the distinct mid-ventral ridge that is seen in cervicals.

Sacral vertebrae. No sacral vertebra has been identified with certainty, but there are three partial vertebrae (D?t, CA-S, S?; Supplementary Data 2) with relatively short square centra and neural arches. These are either sacrals or anterior caudals, but the preservation is poor. Of the three, CA-S is the most likely to be a sacral. The centrum is smoothly rounded in ventral view and the right side of this vertebra preserves the base of a broad lateral process that angles anterolaterally, suggesting it could be the second sacral.

Caudal vertebrae. Only a three, anterior caudal vertebrae have been identified on NMS G.2023.7.1, as identified by their short centra and dorsoventrally compressed transverse processes (e.g. CAa, Fig. 1G–I).

Pectoral girdle and forelimb.

This region is represented by a partial right coracoid and the two humeri (Fig. 2B–C, Extended Data Fig. 6M). The coracoid is anteroposteriorly elongate, with a short rounded posterior portion and a slender tapering anterior region, although this is unlikely to be complete (Extended Data Fig. 6M). The posterodorsal margin is thickened and bears the coracoid portion of the glenoid surface. Immediately anterior to this is a concave, broken surface (the surface appears broken through a microscope), that represents either the articular surface for an unfused scapula, or the broken base of the scapula. Anteroventral to this, there is a large supracoracoid foramen. The margin of the coracoid anterior to the ?scapular facet is much thinner

and is gently concave. Given the position of the glenoid, ?scapular facet, and supracoracoid foramen, this concave margin is likely the ventral border of the scapulocoracoid fenestra, but damage to the anterior coracoid blade makes it impossible to determine whether primary and/or secondary coracoid emarginations were present.

Both humeri are preserved, missing their distal portions (Figs 2B–C). The preserved portion of the right humerus is 9.8 mm long and that of the left humerus is 10.2 mm, but as both bones are missing at least half of the distal shaft our estimated original length is 18–20 mm, which is 4.9 mean dorsal vertebral lengths. The right humerus has its proximal epiphysis fully ossified but slightly detached, demonstrating that it not yet fused to the shaft at the time of death. The humeral head is depressed with a convex terminal head (on the epiphysis) that is medially offset from the long axis of the shaft.

Pelvic girdle and hind limb.

The right ilium and a fragment of right pubis are preserved and give the impression of a relatively gracile pelvis (Fig. 2A). Both bones have intact articular surfaces where they meet at the acetabulum, showing that the pelvic elements were not co-ossified at the time of death.

The ilium is a long slender bone (distance from posterodorsal apex to the intersection of the pubic and ischiadic articular surfaces = 15.1 mm, or 3.87 mean dorsal vertebral lengths). It shows no development of a preacetabular process or tubercle, nor an anterior pubic process, and the relatively small acetabular section grades into the iliac blade without any sharp distinction between the two regions. The blade tapers at the tip and has a low crest along most of its dorsal margin except towards the tip. It is mediolaterally flattened for most of its length, but the distal fifth expands mediolaterally, and flattens dorsoventrally. The medial articular surfaces for the sacral ribs are placed on the posterior half of the blade.

A fragment of the right pubis is preserved in partial articulation with the right ilium (Fig. 2A). The proximal head is subdivided unequally into an articular surface for the ilium and a smaller surface, almost at right angles to the first, for the missing ischium. The posterior margin of the pubic neck is complete and gently curved, forming part of a thyroid fenestra. The broken end of the pubic blade is notched by the pectineal (=obturator) foramen.

The right hind limb is represented by the right femur in two pieces, missing its distal end, and a complete right tibia (Fig. 2E–G). An almost complete left femur is also preserved, at the edge of the block furthest from the braincase (Figs 1B,2D). The proximal part of a possible fibula is also preserved but yields no significant anatomical detail. In all proportions, the hind limb elements fall within the range of normal limbed lizards when compared to mean dorsal vertebral length.

The left femur is 18.9 mm long as preserved without the proximal and distal ends, and may therefore have been 20–21 mm originally, making it 4.84 (preserved portion) – 5.38 (maximum estimated original length) dorsal vertebral lengths. The preserved portion of the right femur is ~15.3 mm long, missing the distal end.

The femur is robustly built and, at most, only slightly curved rather than sigmoid. The proximal and distal ends are set at roughly 45 degrees to one another, separated by a shaft that is round in cross-section. The large terminal femoral head forms a somewhat flattened ellipsoid in cross-section (indicating movement largely in the horizontal plane), and a large internal trochanter signalling strong muscle attachments.

The tibia is damaged at both ends (Fig. 2E). Like the femur, it is robust, with a broad and deep proximal head and a narrower distal end that is roughly half the width of the proximal head. As preserved, the bone is roughly 11 mm in length, but may originally have been ~14 mm as the distal part of the shaft is just beginning to widen out toward the distal head. The tibia is roughly 74% of femoral length.

One metapodial bone in two parts and three phalanges are distributed on the block (Extended Data Fig. 6N–T). Whether they belong to the manus or pes is uncertain but they lie close to the hind limb bones suggesting they are likely pedal elements. Of the phalanges, one is an ungual. It is relatively short and deep with a strongly concave proximal articular surface for the penultimate phalanx, but without prominent extensor or flexor tubercles.

Part C. Osteohistology

Humerus. The humerus has a remarkably thick cortex that preserves the entire record of growth for this element (Fig. 2H–I). The medullary cavity has drifted slightly to partially obscure parallel-fibered bone that represents the earliest record of growth. More peripherally, the cortex is composed of lamellar bone that is interrupted by nine LAGs. The spacing of the growth marks is not consistent throughout the cortex, similar to the femur. Five regularly spaced growth marks are present in the deepest cortex and bound a thick band of lamellar tissue in the middle cortex. More peripherally, four LAGs show a slight reduction in spacing along the sub-periosteal edge. Osteocyte lacunae are not as densely distributed when compared to the femur but remain lenticular and well-aligned with the lamellar mineral fibre orientation. As in the femur, the cortex is almost entirely avascular except for one small, simple vascular canal located in the dorsal quadrant.

Femur. The femur is composed of lamellar bone with at least six growth marks (Fig. 2K–J). The earliest record of growth has been removed due to endosteal remodelling and expansion of the medullary cavity. Multiple generations of deep cortical remodelling are evident in the slightly distally located thin section that shows remodelled trabecular structures and large erosions cavities along the perimedular edge. Five LAGs (line of arrested growth) are visible in this thin section. In the slightly more proximally located thin section, the cortex is thicker and preserves six LAGs, including double LAGs. The spacing of these growth marks is not consistent throughout the cortex; two LAGs are present in the deepest cortex and bound a mid-cortical region of uninterrupted lamellar bone. More peripherally, four LAGs show a reduction in spacing along the sub-periosteal edge, indicating that this individual had multi-phased growth throughout ontogeny and was at or near maturity at death.

References

67. Evans, S. E. & Waldman M. in The Continental Jurassic Vol. 60 (ed. Morales, M.) 219–226 (Museum of Northern Arizona Bulletin, 1996).

68. LeBlanc, A. R. H. et al. Tooth attachment and pleurodont implantation in lizards: Histology, development, and evolution. J. Anat. 238, 1156–1178 (2021).
